# Enhancing the Performance of Metal-Supported Solid Oxide Fuel Cells via Infiltration with an Aqueous Solution of Metal Nitrate Salts

**DOI:** 10.1021/acsami.4c19043

**Published:** 2025-02-03

**Authors:** Aroosa Javed, Daniel Sikstrom, Yoshihisa Furuya, Nilesh Dale, A. Mohammed Hussain, Venkataraman Thangadurai

**Affiliations:** † Department of Chemistry, University of Calgary, Calgary, Alberta T2N 1N4, Canada; ‡ Nissan Technical Center North America (NTCNA), Farmington Hills, Michigan 48331, United States; § School of Chemistry, University of St Andrews, St Andrews KY16 9ST, U.K.

**Keywords:** cathode materials, infiltration, solid oxide fuel cell, praseodymium oxide, oxygen reduction reaction, distribution function of relaxation times, electrochemical impedance spectroscopy

## Abstract

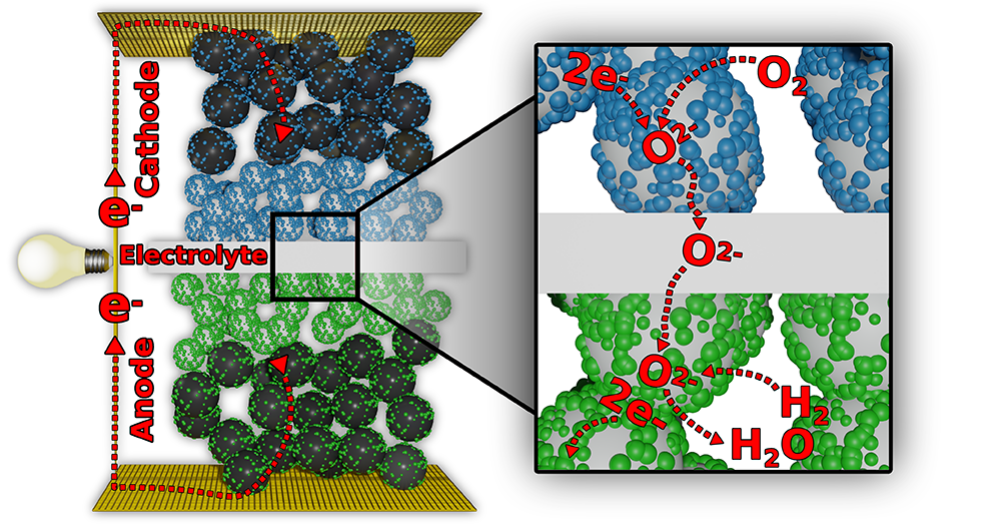

The infiltration technique is a cost-effective method to develop nanostructured electrodes that can accelerate sluggish oxygen reduction reaction (ORR) and enhance the electrochemical performance of solid oxide fuel cells (SOFCs) at intermediate temperatures (600–800 °C). For metal-supported SOFCs, identifying a highly efficient ORR catalyst is an ongoing challenge due to lower temperature operation. In this work, nanostructured praseodymium oxide (PrO_*x*_) and multiphase heterostructures containing perovskites with the nominal composition of Nd_0.6_Sr_0.4_CoO_3−δ_ (NSC), SrCO_3,_ and CoO have been developed via infiltration into the symmetric metal-supported backbone as binary layer composite, and their electrochemical performance has been investigated. The composite demonstrates enhanced electrochemical performance at various temperatures achieving the lowest polarization resistance (*R*_p_) of 0.05 Ω cm^2^ at 700 °C compared to multiphase NSC alone (0.1 Ω cm^2^) under similar conditions. A distribution function of relaxation time (DFRT) analysis using impedance spectroscopy genetic program (ISGP) was carried out to study different electrochemical processes. PrO_*x*_ significantly improves the processes involved in the ORR. The full cell performance of the composite electrode achieves a peak power density (PPD) of 329 mW·cm^–2^ at 700 °C in 3%H_2_O/H_2_ as fuel.

## Introduction

Solid oxide fuel cells (SOFCs) are high-temperature energy conversion devices that produce electricity directly from a gaseous fuel by electrochemically combining with an oxidant.^1−4^ SOFCs are highly attractive due to their high efficiency, fuel flexibility, and low pollutant emissions.^2^ However, the high operating temperatures (800–1000 °C) present several material challenges, including degradation, sealing issues, and high costs.^5,6^ These challenges currently make SOFCs less competitive compared to other energy conversion technologies. Current research on SOFCs has mainly concentrated on reducing SOFC operating temperatures to an intermediate temperature (IT) range (600–800 °C), which brings many advantages such as low cost, improved durability, and broader selection of materials.^7,8^ The performance of IT-SOFCs is limited by the electrode performance, necessitating further research to develop suitable electrode materials that can enhance the performance of IT-SOFCs.^6^ Optimizing electrode performance requires not only the appropriate material composition but also effective electrode engineering to improve the transport of gases, ions, and electrons. Electrode processing parameters such as sintering temperature, duration, and the operating environment play a key role in determining the final microstructure and composition of the electrode.^9^

The standard method for preparing electrodes in an anode-supported SOFC involves the preparation of electrode slurry by mixing the electrode material with an ink and then depositing it on electrolyte using slurry coating, tape casting, or screen printing method.^6,10^ In these methods, the sintering temperature must be optimized to make a strong contact between the electrode and the electrolyte and to minimize the interfacial reactions. However, cathode materials prepared through these methods typically have a low surface area, resulting in higher area-specific resistance for ORR at lower temperatures.^9^ Recently, there has been growing interest in the fabrication of nanoscale electrodes with high electrocatalytic surface area and improved electrochemical ORR performance. The techniques used for the fabrication of these nanoscaled electrodes include thin film deposition, infiltration, and ex-solution.^11^

Infiltration is a cost-effective method to improve the electrocatalytic activity and stability of electrode materials for the IT range. In this method, a porous backbone, such as gadolinium-doped ceria (GDC) or a porous metal support (MS) is prepared, and the catalyst is introduced into the backbone by infiltration.^12,13^ The desired phase of the nanostructured catalyst is obtained after subsequent thermal treatment.^2^ In recent years, various perovskite-based electrode materials have been prepared by this method, which show promising ORR performance.^14−17^ The infiltration is typically carried out in metal-supported cells, which are considered ideal candidates for IT-SOFCs, as they contain thin electrolytes sandwiched between low-cost stainless steel support and show reduced ohmic losses at ITs.^18−20^ They also provide improved mechanical strength, high thermal conductivity, and excellent tolerance to redox and thermal cycling.^21−23^ Since the highest efficiency loss in IT-SOFCs is caused by the sluggish kinetics of ORR at the cathode, most research studies have focused on studying the performance of different infiltrates as cathode catalysts.^16,24^

Conventional cathode materials, such as (La,Sr)MnO_3_ (LSM) and (La,Sr)(Co,Fe)O_3_ (LSCF), require high temperature (1000–1200 °C) sintering, which make them unsuitable for MS-SOFCs. At high temperatures (>900 °C), the oxidation of the MS damages the structural integrity of the cell.^23^ LSM cathodes were generally sintered in N_2_ at 1100 °C to avoid oxidation of the MS; however, none of these compositions showed good ORR performance at ITs.^25^ Additionally, LSCF cathodes readily react with the Cr present in the MS and reduce the catalytic activity of the cathode.^26^ Lawrence Berkeley National Laboratory (LBNL) has developed an infiltration method to overcome the challenges related to cathode fabrication.^27−29^ In this method, precursor salts of the metals comprising the catalyst are impregnated into the porous electrode backbone as infiltrates. These infiltrates decompose at 400–600 °C to produce the intended oxide catalyst, hence avoiding the deleterious reactions between the catalyst and other cell components, such as electrolyte and the MS. The porous stainless steel current collector is coated with the catalyst during the infiltration process.^30^ Catalyst coatings formed by the rare earth elements can improve the oxidation resistance of the current collector and prevent Cr evaporation from the MS.^31,32^

Praseodymium oxide (PrO_*x*_) is a popular cathode material due to its mixed ionic and electronic conducting properties.^33,34^ PrO_*x*_, when combined with other catalysts such as (La_0.6_Sr_0.4_)_0.95_Co_0.2_Fe_0.8_O_3−δ_ (LSCF) and SrTi_0.3_Fe_0.55_Co_0.15_O_3−δ_ (STCF), serves as a cathode material to improve their electrochemical performance at ITs.^34^ In this study, we report the electrochemical performance of two cells that were infiltrated by using aqueous solutions of metal nitrate salts. One cell was infiltrated with an aqueous solution of metal nitrate salts corresponding to the nominal composition of Nd_0.6_Sr_0.4_CoO_3−δ_ (NSC). The other cell was initially infiltrated with an aqueous solution of praseodymium nitrate to form praseodymium oxide (PrO_*x*_), followed by a second infiltration step using NSC solution to develop a binary layer composite of PrO_*x*_-NSC inside the porous backbone. The infiltrates are introduced layer-by-layer so that each layer contains a specific functionality to improve the ionic and electronic conductivity of the electrode.^35^ The impedance results were analyzed by using a distribution of relaxation time (DRT) analysis using the impedance spectroscopy genetic program (ISGP) to carefully investigate the role of PrO_*x*_ in performance enhancement.

## Experimental Section

### Cell Fabrication

MS cells used in this work are originally developed by LBNL.^29,36−39^ Briefly, green cells are assembled by laminating individual 10Sc1CeSZ (DKK, Japan) and stainless steel (P434L alloy, water atomized, Ametek Specialty Metal Products) layers prepared by tape-casting. The layers are prepared with poly(methyl methacrylate) poreformer beads (Esprix Technologies) and a water-based tape-casting binder. The resulting symmetric-structure MS-SOFCs are laser-cut (Full Spectrum Laser) from the laminated tape-cast layers, and the edges are cleaned with an air duster to remove any loose particles. Cells are then debonded by firing in air in a box furnace at 500–600 °C for 1 h with 3 °C min^–1^ heating rate to remove the binder and poreformer. The cells are then sintered at 1300–1350 °C for 2 h in a tubular furnace while flowing 10% hydrogen in argon. The resulting cells are 25 mm in diameter with ∼5 cm^2^ active area; 200 μm thick, porous MS; 25 μm thick, porous cathode and anode backbones; and ∼7 to ∼12 μm thick 10Sc1CeSZ electrolyte. Extensive details about the optimization of each cell layer are described elsewhere.^37,40^

### Catalyst Precursors and Cell Infiltration

The precursor 3 M solutions of metal nitrate salts corresponding to the nominal composition of Nd_0.4_Sr_0.6_CoO_3_ (NSC) are prepared by dissolving stoichiometric quantities of metal nitrates in deionized water and Triton-X 100 (10 wt %) (Sigma-Aldrich). For the anode solution, stoichiometric quantities of the precursors are dissolved in water and Triton-X-100 to form a 20% Gd-doped ceria/Ni (Ni-GDC, 60/40 vol %) anode catalyst solution. Triton-X 100 is used as a surfactant to disperse the precursors uniformly. The mixture is stirred at room temperature for 1 h. The infiltration solution is injected into porous frameworks with a pipet, and then the cell is vacuumed for 15 min to deposit the infiltration solution into the channels. Cells are infiltrated three times to achieve infiltrate loading (∼5 wt %), which is determined by weight difference before and after infiltration. Multiple infiltrations provide the opportunity to fire the same catalyst composition at different temperatures. After the first infiltration, the cell is heated to 800 °C for 1 h to provide a good electronic network. The subsequent infiltrations are followed by heating at 600 °C to form smaller particles with a high surface area. To prepare the composite electrodes, PrO_*x*_ is infiltrated first, followed by infiltration with NSC solution.

### Characterization

The crystal structures of the powders are characterized by powder X-ray diffraction (PXRD) using a Bruker D8 advance diffractometer in Bragg–Brentano geometry (40 kV, 40 mA) with Cu Kα radiation (λ = 1.5418 Å). The XRD scanned the sample with a speed of 5 °C min^–1^ at a step of 0.01° and in 2θ range of 20–80°. For the microstructure analysis, cells are mounted in epoxy, cut, and polished to prepare them for scanning electron microscope (SEM). The microstructure images of the infiltrated cells are collected using a Quanta FEG250 SEM instrument equipped with an Everhart Thornley Detector (ETD) and Backscatter Electron Detector (BSD) at a voltage of 15 kV. (Thermo Fisher). X-ray microanalysis is done using a Bruker Quantax system consisting of a Quantax 5030 silicon drift detector, Bruker SVE III pulse processor, and Bruker Esprit v2.3 software running on a Windows platform.

### Electrochemical Analysis

A Solartron 1287/1255 impedance analyzer is used to acquire electrochemical impedance spectra (EIS) for half and/or full cells in the frequency range of 10^–1^–10^6^ Hz at an ac amplitude of 10 mV under open circuit voltage (OCV) in ambient air. The temperature dependence of *R*_p_ from 600 to 700 °C is studied at intervals of 25 °C. For full cell testing, the Pt mesh is spot-welded on each side of the cells to make electrical connections to Pt wires connected to the electrochemical testing interface. Infiltrated cells are mounted on an alumina-based reactor and Ceramabond is used as a sealant to seal the cell. Mass flow controllers (MFCs) controlled gas flow, fixing the flow rate of hydrogen and air to 200 and 100 sccm, respectively. For different oxygen partial pressure (pO_2_) experiments, the total flow rate is 50 sccm and the pO_2_ is changed by changing the flow rate of oxygen and nitrogen by their respective MFCs. Solartron 1287/1255 impedance analyzer is used to record current density–voltage–power density (*I*–*V*–*P*) curves along with EIS at 700, 650, and 600 °C by using humidified (3 vol % H_2_O) hydrogen as the fuel and air as the oxidant on anode and cathode sides, respectively.

The EIS of the half cells is analyzed through ISGP. This program avoids the overfitting of the data by using discrepancy-complexity approach and provides accurate results which originate from the experimental data.^12^Equation 1 shows the relationship between the measured impedance and DRT.^41^
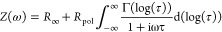
1where *Z* is the impedance, *R*_∞_ is the ohmic resistance, *R*_pol_ is the total polarization resistance (*R*_p_), Γ is the distribution of resistances characterized by their relaxation times, τ is the relaxation time, and ω is the angular frequency. This program generates peaks representing different processes as a function of time domain. Each peak has a specific area that on multiplication with total resistance gives the *R*_p_ of each process.

## Results and Discussion

### Catalyst Structure and Crystallite Size

**Figure 1:**
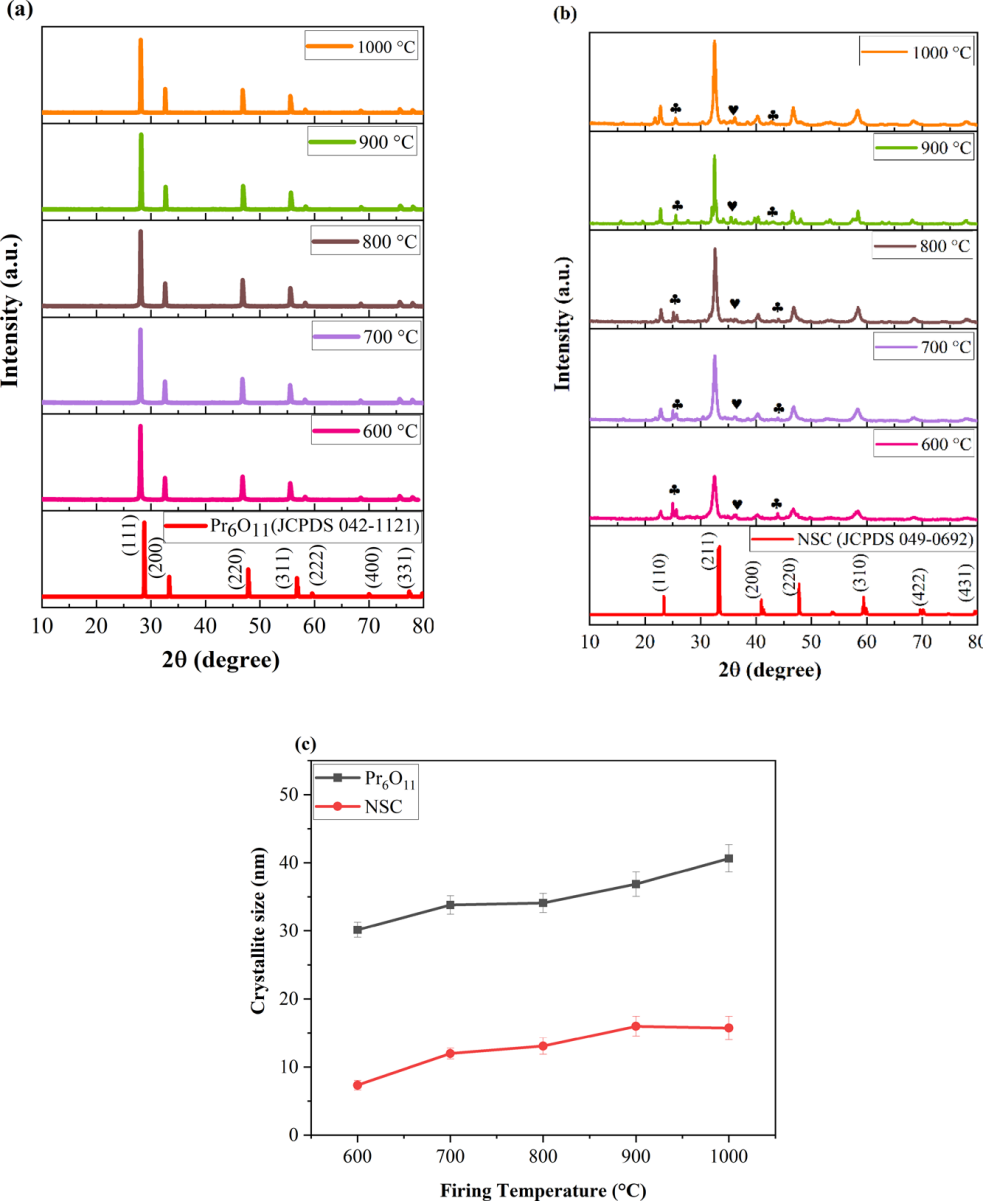
PXRD patterns of the powder calcined at 600–1000 °C in air: (a) PrO_*x*_, (b) NSC, 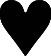 represents the impurity peaks belonging to cubic phase of CoO (Joint Committee on Powder Diffraction Standards (JCPDS) no: 01-089-2803), and 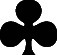 represents the impurity peaks belonging to orthorhombic phase of SrCO_3_ (JCPDS no: 01-084-1778), and (c) crystallite sizes of the powder calcined at 600–1000 °C.

The as-prepared infiltration solutions are heated at 100 °C for 24 h to evaporate the solvent, and the resulting gel is heated at 600–1000 °C in air. Figure 1a,b shows the XRD patterns of the catalyst powders after calcining in the temperature range of 600–1000 °C. Interestingly, no secondary phases are observed and a single phase of Pr_6_O_11_ (Joint Committee on Powder Diffraction Standards (JCPDS) 42-1121) is present at 600–1000 °C. The shifting of the peak in the PXRD pattern indicates a change in lattice parameters. Since the peaks shift toward a lower 2θ value compared to the reference pattern, this indicates an increase in the lattice parameters. For NSC, all the samples calcined from 600 to 1000 °C show the characteristic diffraction patterns of a perovskite-type rhombohedral phase (JCPDS # 00-049-0692). There is some peak splitting observed in the XRD pattern which may be attributed to the rhombohedral phase.^42,43^ Small amounts of impurity phases such as SrCO_3_ (JCPDS # 01-084-1778) and CoO (JCPDS #01-089-2803*)* are also present, which persisted even at higher temperatures. The phase fraction of CoO and SrCO_3_ was calculated from the XRD intensity ratio; *I*_i_/*I*_total_ where *I*_i_ is the intensity of impurity phase such as SrCO_3_ or CoO and *I*_total_ is the sum of intensities of all the phases present.^44^ The phase fraction of SrCO_3_ decreased from 23.32 to 8.52%, whereas the phase fraction of CoO decreased from 9.62 to 7.2% on increasing the temperature from 600 to 1000 °C. The average crystallite size of Pr_6_O_11_ and NSC were also calculated with the Scherrer equation, which showed an increase in crystallinity with firing temperature. The obtained crystallite size values range from 30.15 to 40.64 nm for PrO_*x*_ and 7.34–15.73 nm for NSC (Figure 1c).

### Electrochemical Characterization

**Figure 2:**
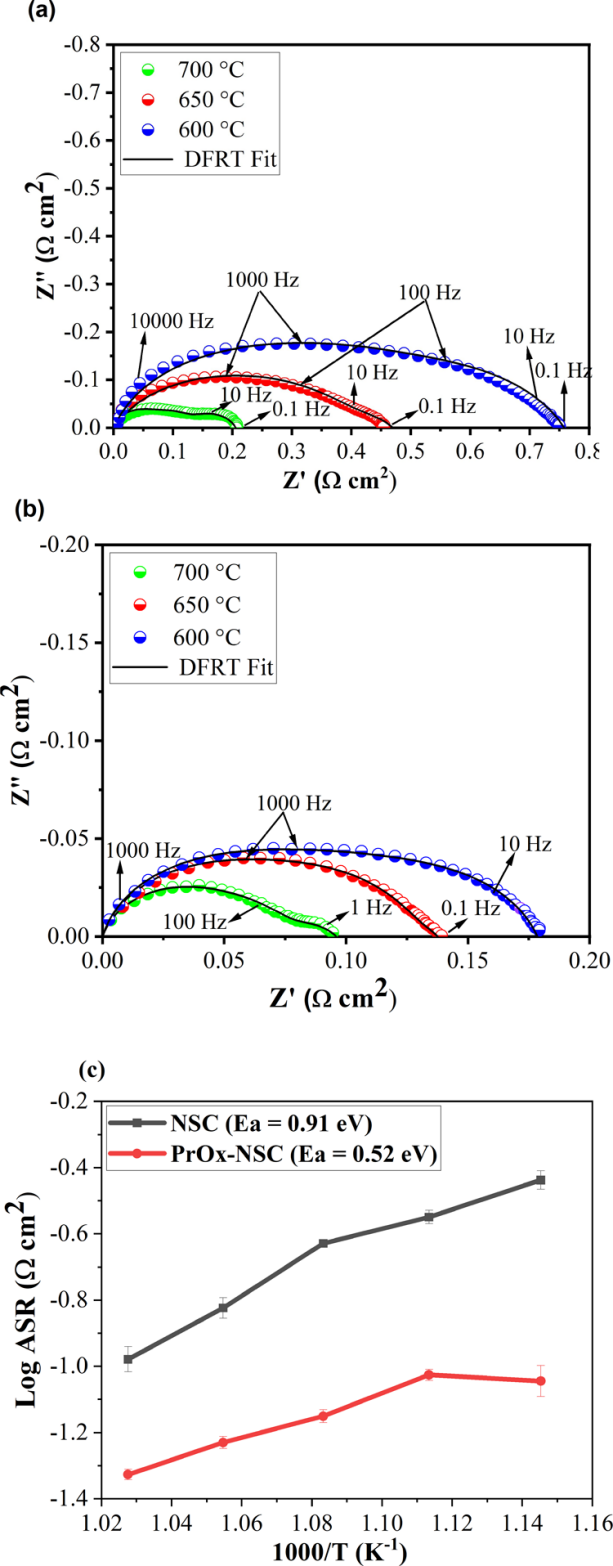
*R*_s_-corrected EIS curve of cells infiltrated with (a) NSC, (b) PrO_*x*_-NSC measured under OCV at 600, 650, and 700 °C in ambient air, and (c) Arrhenius plot of the symmetrical cells in ambient air at 600–700 °C.

The catalytic activity of the infiltrated symmetric cells is examined in air at 600–700 °C under OCV (Figure 2). The series resistance (*R*_s_) is normalized to zero to show a comparison of the *R*_p_ of the electrodes. Nyquist plots of cells infiltrated with NSC and PrO_*x*_-NSC show a change in the *R*_p_ of the infiltrated electrodes at various temperatures (Figure 2). The *R*_p_ values at 700, 650, and 600 °C are 0.05, 0.07, and 0.09 Ω cm^2^ for PrO_*x*_-NSC and 0.1, 0.24, and 0.37 Ω cm^2^ for NSC infiltrated cells. The temperature dependence of *R*_p_ clearly shows that the presence of PrO_*x*_ significantly reduces *R*_p_ of the cathode. These findings indicate that PrO_*x*_ acts as a catalyst in cathodic ORR and helps enhance electrochemical performance.^45^ Juanarena et al. reported that the infiltrated Pr_6_O_11_ can form a strong interaction with the electrolyte backbone and therefore extend the triple-phase boundary (TPB) resulting in improved electrochemical performance of the electrode.^46^Figure S1a,b (see Supporting Information) shows that the ohmic resistance of the infiltrated cells also exhibits temperature dependence and changes from 0.77 to 0.94 Ω cm^2^ on changing the temperature from 700 to 600 °C. There are no significant differences in ohmic resistance of both the cells as electrolyte thickness is the same in them.

**Figure 3:**
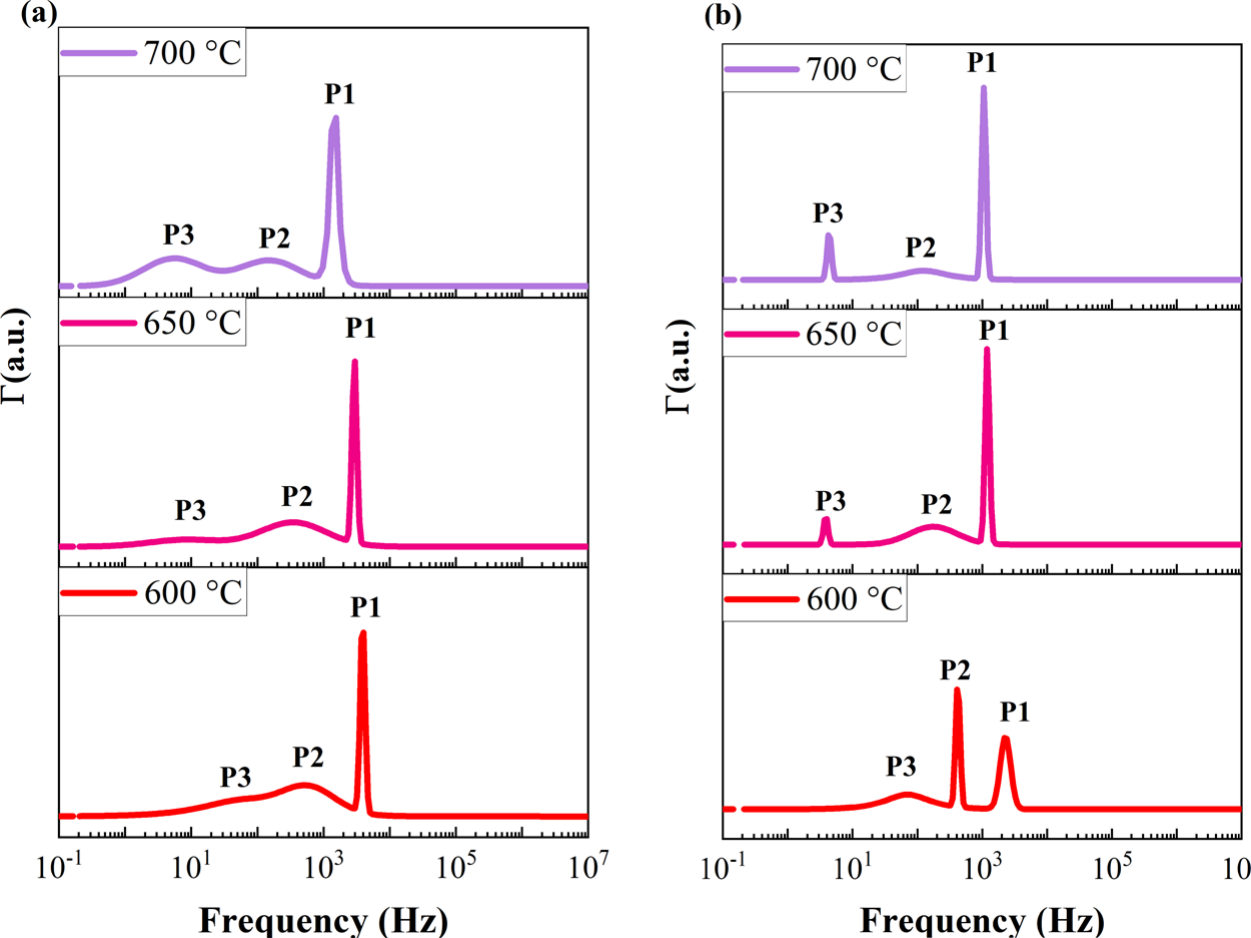
DRT results of the half-cell EIS for cells infiltrated with (a) NSC and (b) PrO_*x*_ -NSC measured in air at 600, 650, and 700 °C.

The DRT analysis of impedance data is used to deconvolute the different electrode reaction processes according to their time scales and to better understand the role of PrO_*x*_ in the cathode performance. The low time constant (high frequency, >10^3^ Hz) region indicates oxygen ions migration at the electrode–electrolyte interface, the intermediate (midfrequency, 10–10^3^ Hz), and high time constant region (low frequency, <1 Hz) indicate the contribution of surface exchange processes (oxygen dissociative adsorption/desorption) and gas diffusion to the total impedance losses, respectively.^34^Figure 3 shows the distribution function of relaxation times (DFRT) plots of NSC and PrO_*x*_-NSC electrodes at 600, 650, and 700 °C obtained using ISGP. The quality of the fitting using DFRT (ISGP) analysis is represented by the residual graph in Figure S2 (see Supporting Information). A smaller residual value (<1.5%) indicates good fitting.

The DFRT data are plotted in the frequency domain *f*, rather than the time domain τ, where *f* = 1/2πτ.^5^ The DFRT plots of both the electrodes show 3 peaks (marked as P1, P2, and P3), indicating that there are at least three independent processes involved in total ORR from 600–700 °C. The resistance contribution of each elementary ORR process to the total *R*_p_ can be calculated by multiplying the area under each peak with the total resistance.^47^ The resistance and time constant have been calculated using the capacitance for each peak (*C*) based on eq 2 as shown below:
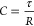
2

A summary of the fitted parameters is given in Table 1. R1, R2, and R3 show the resistance values for corresponding peaks P1, P2, and P3 (Figure 3a,b). As observed in Table 1, the resistance values for all three processes, i.e., R1, R2, and R3 change with temperature, showing that these three processes are related to the electrochemical performance of the ORR. For PrO_*x*_-NSC, the values for R1, R2, and R3 are reduced compared to the resistance values for NSC infiltrated samples; however, significant *R*_p_ reduction is observed for R2 and R3 at all temperatures. These results suggest that PrO_*x*_ infiltration mainly enhances the surface exchange processes, such as oxygen dissociative adsorption/desorption, compared to the charge transfer processes.^48,49^ These findings are also consistent with previous research studies, where a significant improvement in *R*_p_ of the electrodes was reported for PrO_*x*_-infiltrated samples.^33,34,46,50^ Robinson et al. reported that PrO_*x*_ possesses high ionic conductivity, providing additional pathways for oxide ion transport from the backbone surface to the electrolyte and improving the ORR.^33^ Moreover, the cell infiltrated with PrO_*x*_-NSC shows a lower activation energy (*E*_a_) than the cell infiltrated with NSC.

**Table 1:** Fitted Results of ASR for NSC and PrO_*x*_-NSC Cathodes Measured at 600, 650, and 700 °C in Air

composition	*T* (°C)	*R*1 (Ω cm^2^)	*C*1 (mF cm^–2^)	*R*2 (Ω cm^2^)	*C*2 (mF cm^–2^)	*R*3 (Ω cm^2^)	*C*3 (F cm^–2^)	ASR (Ω cm^2^)
NSC	700	0.08	1.67	0.06	26.57	0.06	0.47	0.10
	650	0.14	0.62	0.25	1.95	0.08	0.25	0.24
	600	0.18	0.22	0.29	0.96	0.28	0.01	0.37
PrO_*x*_ -NSC	700	0.05	3.12	0.03	47.79	0.02	3.04	0.05
	650	0.06	2.18	0.07	15.17	0.01	4.68	0.07
	600	0.06	1.10	0.05	8.23	0.07	0.03	0.09

**Figure 4:**
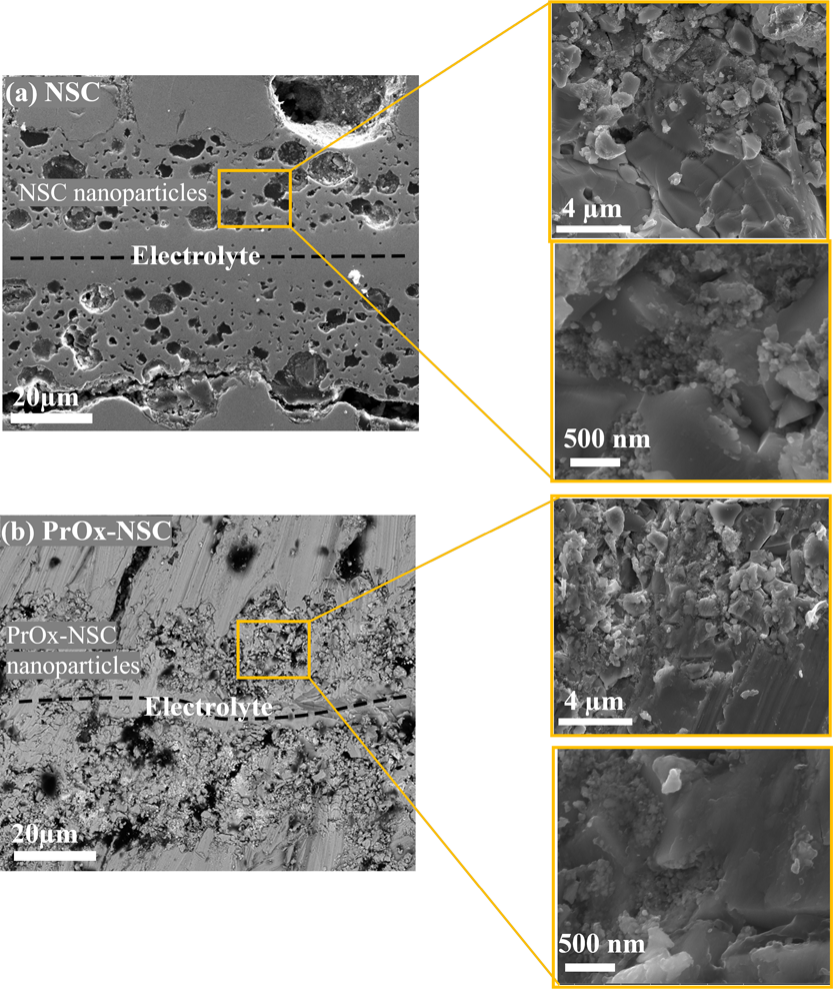
SEM analysis of polished cross sections of MS symmetric cells infiltrated with (a) NSC and (b) PrO_*x*_-NSC after the test in ambient air at 600–700 °C.

Figure 4 shows the cross-sectional SEM micrographs of the half cells infiltrated with NSC and PrO_*x*_-NSC. These cells consist of a dense 7 μm thick 10Sc1CeSZ electrolyte tightly binding with the porous 10Sc1CeSZ layer sandwiched between the porous metal layers. The porous 10Sc1CeSZ layer shows a continuous network with high porosity for efficient infiltrate flow and for sufficient gas diffusion. The strong contact between the porous 10Sc1CeSZ layers and the electrolyte also ensures efficient charge transport between them.^51−53^ The entire porous MS appears to be filled with nanoparticles, and the morphology of the particles in both cells appeared to be the same as they were calcined at the same temperature. The micrographs showed that the nanoparticles were evenly distributed in a porous backbone with particle sizes of 20–100 nm. The cell containing PrO_*x*_-NSC shows sufficient porosity for gas transport. Figure S3 (see Supporting Information) shows the EDX spectra of the infiltrated cells, and the results indicate the presence of Nd, Sr, and Co in the NSC-infiltrated cell, whereas Pr, Nd, Sr, and Co were present in the PrO_*x*_-NSC-infiltrated cell. There results indicate that the nanoparticles were successfully infiltrated in both cells. The EDX spectra also show additional Sc and Zr peaks which arise from the porous backbone of the MS cell.

### Rate-Limiting Steps Involved in ORR

EIS measurements under different oxygen partial pressures (pO_2_) are conducted to investigate the ORR kinetics of infiltrated electrodes at 700 °C. Equation 3 shows that the polarization resistance (*R*_p_) of the electrode varies linearly with pO_2_ as shown below:
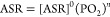
3where *n* provides insight into the specific ORR process and can have different values between 0 and 1, which represent the rate-determining step (RDS) of ORR.^54^ For example, *n* = 1 indicates that diffusion and adsorption of gaseous O_2_ molecules into the pores and on the active sites of the cathode can be the rate-limiting step. An n value of 0.5 shows that the dissociation of adsorbed oxygen molecules into oxygen atoms is the RDS. Similarly, n values of 0.25 and 0 indicate the bulk charge transfer and oxide ion incorporation from the TPB to the electrolyte as the RDS, respectively.^55^

Figures S4a and S5a (see Supporting Information) depict the EIS graphs for the cells infiltrated with NSC and PrO_*x*_-NSC under different pO_2_. The ohmic resistance is subtracted from the spectra to observe the properties of the cathode material better. DRT analysis is performed to determine the rate-limiting steps involved in the ORR for both of the cells (Figures S4b and S5b). The ASR of the infiltrated electrodes shows a gradual decrease with an increase in pO_2_, showing its positive influence on oxygen reduction. Representative DRT curve shows three peaks designated as P1, P2, and P3, which represent the high frequency (HF), medium frequency (MF), and low frequency (LF) resistance peaks, respectively, and indicate three processes involved in the oxygen reduction process. The values and proportions of simulated ASR for three peaks (P1, P2, and P3) are computed to quantify their contribution to the total *R*_p_ (Figures S4c and S5c). The resistance values for all three peaks undergo significant change while transitioning from high pO_2_ to low pO_2_. The *R*_p_ of the infiltrated cells is plotted against pO_2_ to find the value of the slope (*n*) (Figures S4d and S5d). The preliminary results showed that for NSC and PrO_*x*_-NSC-infiltrated cells, *n* values of 0.85 and 0.90 are obtained, which fall between 0.5 and 1. These results indicate that the adsorption and dissociation of oxygen molecules on the electrode surface are the rate-limiting steps for these cathodes.

### Full Cell Performance

**Figure 5:**
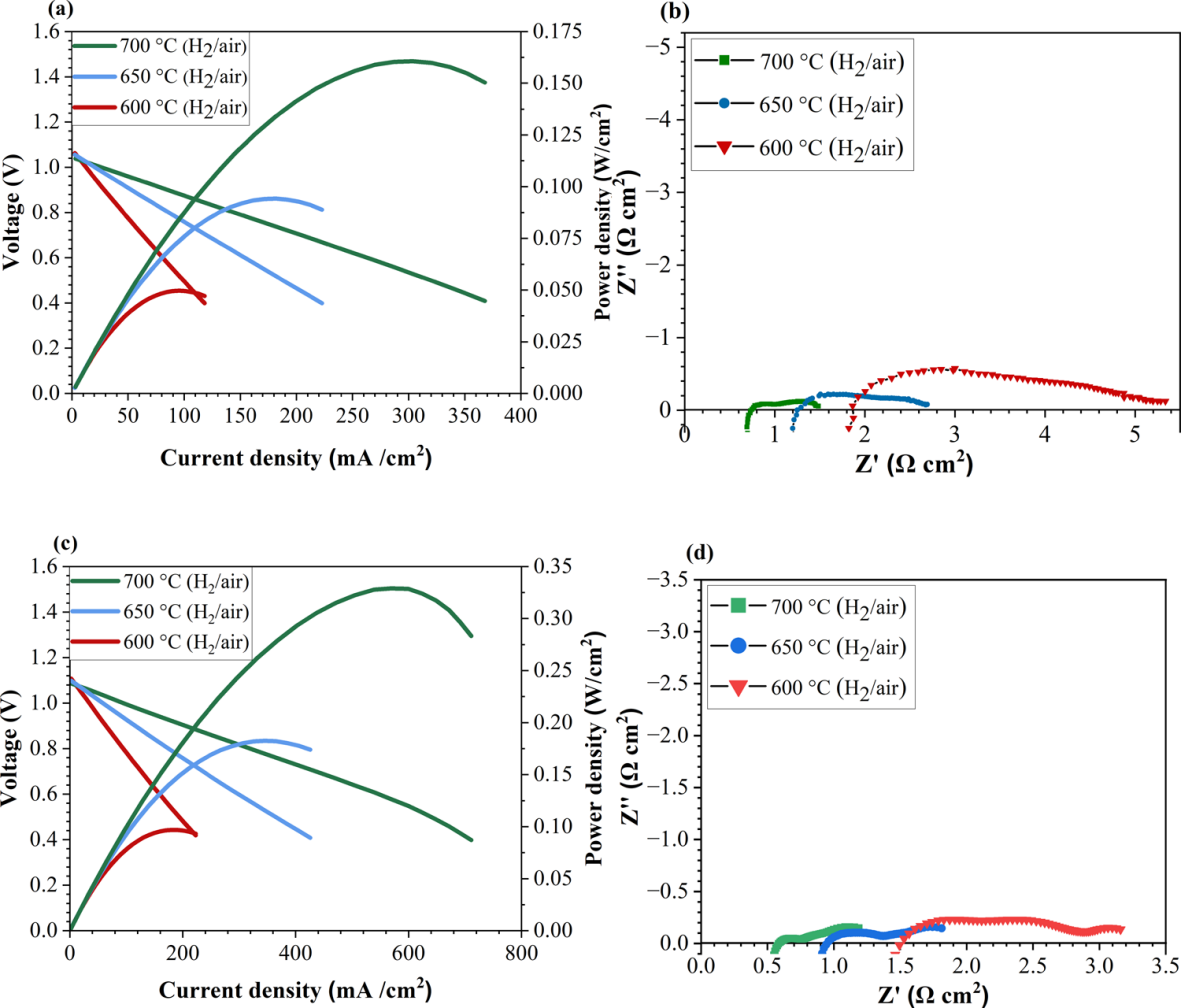
Full cell performance of infiltered cells (a) *I*–*V*–*P* curves of NSC-infiltrated cells, (b) EIS curve of NSC, (c) *I*–*V*–*P* curves of a PrO_x-_NSC infiltrated cell, and (d) EIS curve of PrO_*x*_-NSC measured under OCV at 600, 650, and 700 °C.

**Figure 6:**
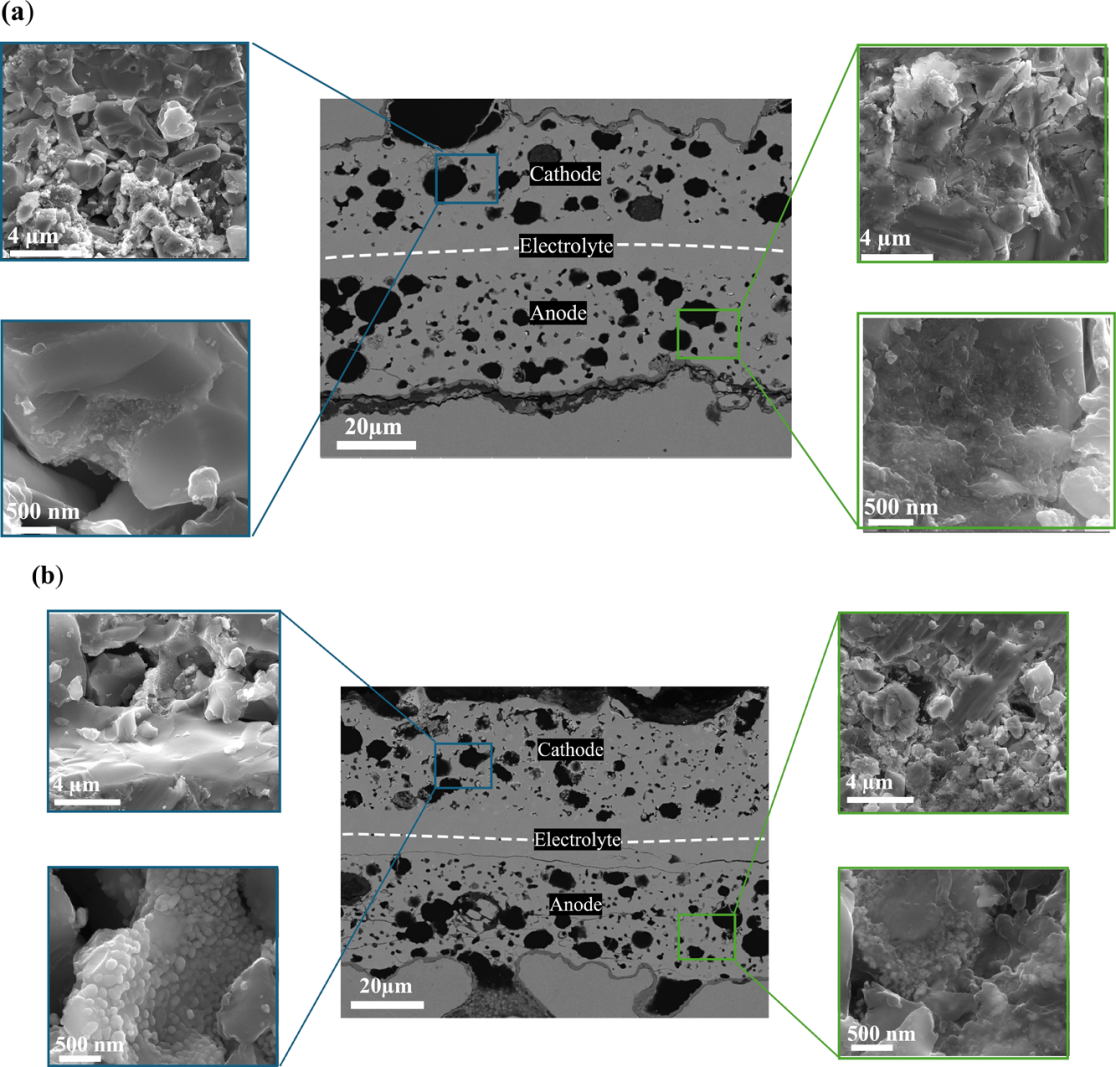
Microstructural SEM images of single cells infiltrated with the Ni-GDC anode and (a) NSC and (b) PrO_*x*_-NSC cathodes after the test in 3%H_2_O/H_2_ at 600–700 °C.

The performance of the composite cathode is tested in a full-cell configuration with the Ni-GDC anode to determine the performance at the full-cell level. Figure 5a,c shows the full cell performance of the cell at various temperatures. The current density–voltage-power density curves show that the peak power density (PPD) of a single cell increases to 329 mW/cm^2^ for the PrO_*x*_-modified cell, which is higher than a bare NSC-infiltrated cell (160 mW/cm^2^ at 700 °C). EIS is also measured for both cells under an OCV (Figure 5b,d). It is found that the *R*_p_ significantly reduced from 0.73 to 0.60 Ω cm^2^ at 700 °C for the PrO_*x*_-modified cell showing the effect of PrO_*x*_ infiltration in enhancing the electrocatalytic performance of the cell. Table 2 shows a comparison of PPD of MS cells with various infiltrated catalysts reported in the literature.^35,36,39,56−62^ Although the PPD of our catalyst is not the highest compared to other catalysts; however, it still shows comparable performance to most of the YSZ and ceria-based electrolytes reported in the literature. Figure S8 (see Supporting Information) shows the short-term performance of PrO_*x*_-NSC infiltrated single cell, which was operated for 65 h at a current density of 0.2 A/cm^2^ and at 700 °C. Rapid voltage drop from 0.89 to 0.60 V was observed corresponding to the degradation rate of ∼33%/65 h. The degradation rate is much higher than that of La_0.6_Sr_0.4_Fe_0.9_Sc_0.1_O_3_d_ (LSFSc)-infiltrated cells which showed a degradation rate of ∼20%/150 h when operated at a current density of 0.86 A/cm^2^ at 700 °C.^63^ One of the possible reasons for rapid degradation can be Ni coarsening, which was observed even in SEM analysis (Figure 6). Thus, degradation study is further needed to understand the mechanism in metal-supported cells.

**Table 2:** Comparison of Electrochemical Performance of Cathode Materials for MS-SOFCs^35,36,39,56−62^

cell configuration	electrolyte thickness (μm)	fabrication method	ASR (Ω cm^2^) *T* (°C) (EIS method and conditions)	PPD (mW/cm^2^), *T* (°C)	ref
PrO_*x*_-NSC/10Sc1CeSZ/Ni-GDC	7	infiltration	0.60 (700) OCV	329 (700)	this study
LSM/YSZ/Ni-YSZ	0.15	infiltration	∼0.30 (700)	332 (700)	(36)
LSM-YSB/YSZ/Ni-SDC	48 ± 2	screen printing (cathode)/infiltration (anode)	0.51 (700)	246 (700)	(57)
ESB-Ag_2_O/YSZ/Ni-YSZ	400	screen printing	0.18 (700) OCV	380 (700)	(59)
LSFSc/YSZ/Ni	30	infiltration	1.32 (700), OCV	418 (700)	(62)
LSM-SDC-PrO_*x*_/10Sc1CeSZ/Ni-SDC	7–12	infiltration	0.19 (700), 0.7 V	1120 (700)	(35)
LSCF/GDC/YSZ/LSTN-YSZ/STS434L	5	screen printing	0.08 (650)	176 (650)	(61)
PrO_*x*_-/10Sc1Ce/Ni-SDC		infiltration	∼0.85 (700), OCV	520 (700)	(39)
LSCF/YSZ/LST	1.5	screen printing (cathode)/Ni infiltration (anode)	0.12, OCV	340 (750)	(60)
SFMO-YSZ/YSZ/Ni-YSZ	16	impregnation	0.13 (700) OCV	221 (700)	(58)
SBSCO/SSZ/Ni-SDC	250	infiltration	0.11 (700) OCV	1250 (700)	(56)

Figure 6 shows the cross-sectional SEM micrographs of a single MS-SOFC cell consisting of porous 10Sc1CeSZ impregnated cathode, dense 10Sc1CeSZ electrolyte, and a porous 10Sc1CeSZ impregnated anode substrate. The dense electrolyte layer and the porous layer seem to be well-connected, and no visible cracks are observed. The SEM results showed that the nanoparticles were uniformly distributed over the porous metal–support and no obvious elemental diffusion was observed at the interface. The magnified images of NSC and PrO_*x*_-NSC infiltrated cells show uniform distribution of nanoparticles which are also well intraconnected on internal surfaces of porous 10Sc1CeSZ layers. The infiltrates are deposited throughout the thickness of the substrate indicating that there were enough capillary forces to propel the infiltrate solution to the electrode/electrolyte interface. Higher magnification SEM micrographs show the particle diameter of 20–100 nm, which indicates a larger surface area of infiltrated electrode materials than conventional electrodes resulting in fast fuel oxidation and oxygen reduction.^64^ From the SEM images, it can be seen that the microstructure looks quite dense on the anode side, which can be due to Ni coarsening.

The EDX analysis of the single cells is carried out to confirm the presence of specific elements in the porous 10Sc1CeSZ backbone (Figures S6 and S7). All elements in NSC and PrO_*x*_-NSC samples are distributed in the porous backbone. There were some impurity peaks of Ni on the cathode side in the NSC-infiltrated cell, which can be due to the infiltrate crossover.

## Conclusions

In this study, infiltrated catalysts with nominal compositions of Nd_0.6_Sr_0.4_CoO_3−δ_ (NSC) and PrO_*x*_-Nd_0.6_Sr_0.4_CoO_3−δ_ (PrO_*x*_-NSC) are deposited into porous metal-supported backbone via infiltration and their electrochemical performance is studied. Half-cell studies showed that the cell infiltrated with PrO_*x*_-NSC shows a significant reduction in the *R*_p_ at all temperatures which is attributed to the extension of the triple phase boundary resulting from PrO_*x*_ infiltration. EIS results, along with DRT, demonstrate that the presence of PrO_*x*_ significantly improves the kinetics of ORR resulting in improved electrochemical performance. The PPD of PrO_*x*_-NSC infiltrated cell reaches about 329 mW/cm^2^ at 700 °C, which is almost 2 times higher than that of single-cell without PrO_*x*_. These results demonstrate that introducing composite electrodes into porous backbone can significantly enhance the SOFC performance compared to the pristine cell. Moreover, using this approach, the cathode *R*_p_ can be significantly reduced, which can also reduce the operating temperature of SOFC.

## Supporting Information Available

EIS spectra of the cells infiltrated with NSC and PrO_*x*_-NSC under open circuit voltage at 600, 650, and 700 °C tested in ambient air, residual Vs. log10(ω) plot to compare the compatibility of measured impedance with the fitted data, SEM images and EDX spectra of half cells infiltrated with NSC and PrO_*x*_-NSC, EIS spectra and DRT analysis of NSC and PrO_*x*_-NSC infiltrated cell in different pO_2_ at 700 °C to determine the rate-limiting steps, SEM images and EDX spectra of single cells infiltrated with NSC and PrO_*x*_-NSC as the cathode and Ni-GDC as the anode, and short-term stability test of full cell using PrO_*x*_-NSC infiltrates (PDF)
