# Supplementary material for: Enhancing the Performance of Metal-Supported Solid Oxide Fuel Cells via Infiltration with an Aqueous Solution of Metal Nitrate Salts
Source: ACS Appl Mater Interfaces. 2025 Feb 3;17(6):9305–14. doi: 10.1021/acsami.4c19043 (PMC11826904; doi:10.1021/acsami.4c19043)
Supplement: Supplementary file 1 — am4c19043_si_001.pdf [file am4c19043_si_001.pdf]

Supporting Information

Enhancing the Performance of Metal-Supported Solid Oxide Fuel Cells via Infiltration with an Aqueous Solution of Metal Nitrate Salts

Aroosa Javed[a], Daniel Sikstrom[a], Yoshihisa Furuya[b], Nilesh Dale[b], A. Mohammed Hussain[b*] and Venkataraman Thangadurai[a,c,**]

a. University of Calgary, Department of Chemistry, Calgary, Alberta, T2N 1N4, Canada

b. Nissan Technical Center North America (NTCNA), Farmington Hills, Michigan 48331, USA

c. School of Chemistry, University of St Andrews, St Andrews, Fife, KY16 9ST, UK

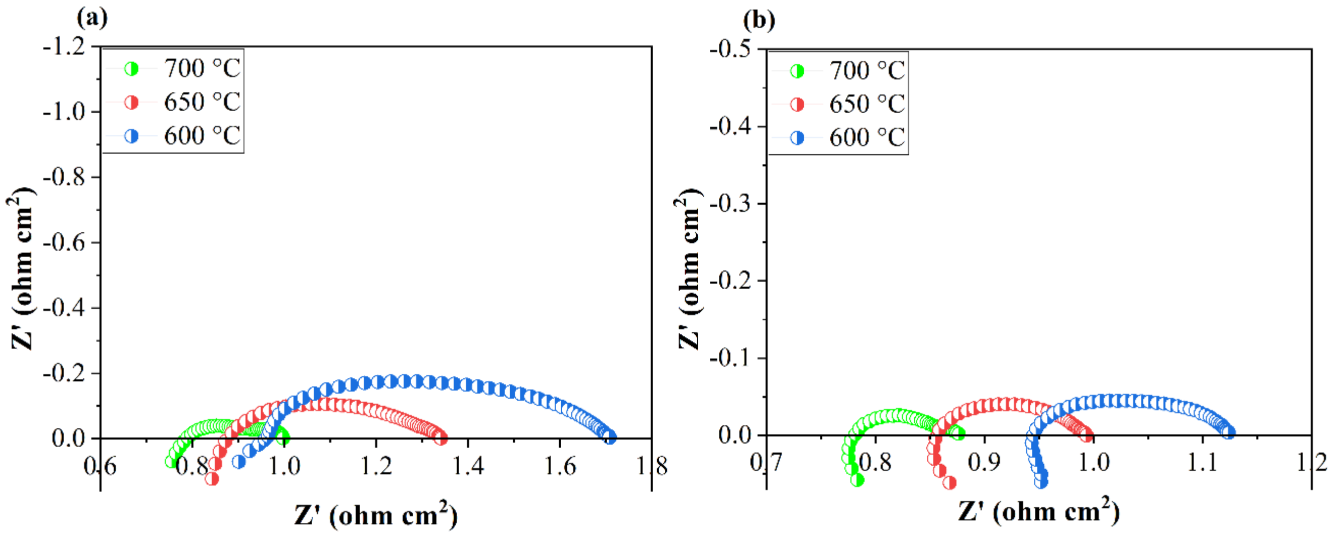

Figure S1. EIS curve of cells infiltrated with (a) NSC (b) PrO_x -NSC measured under OCV at 600, 650 and 700 °C in ambient air.

* Corresponding author.
** Corresponding author.
Email addresses: vthangad@ucalgary.ca; vt36@st-andrews.ac.uk; hussain.jabbar@nissan-usa.com

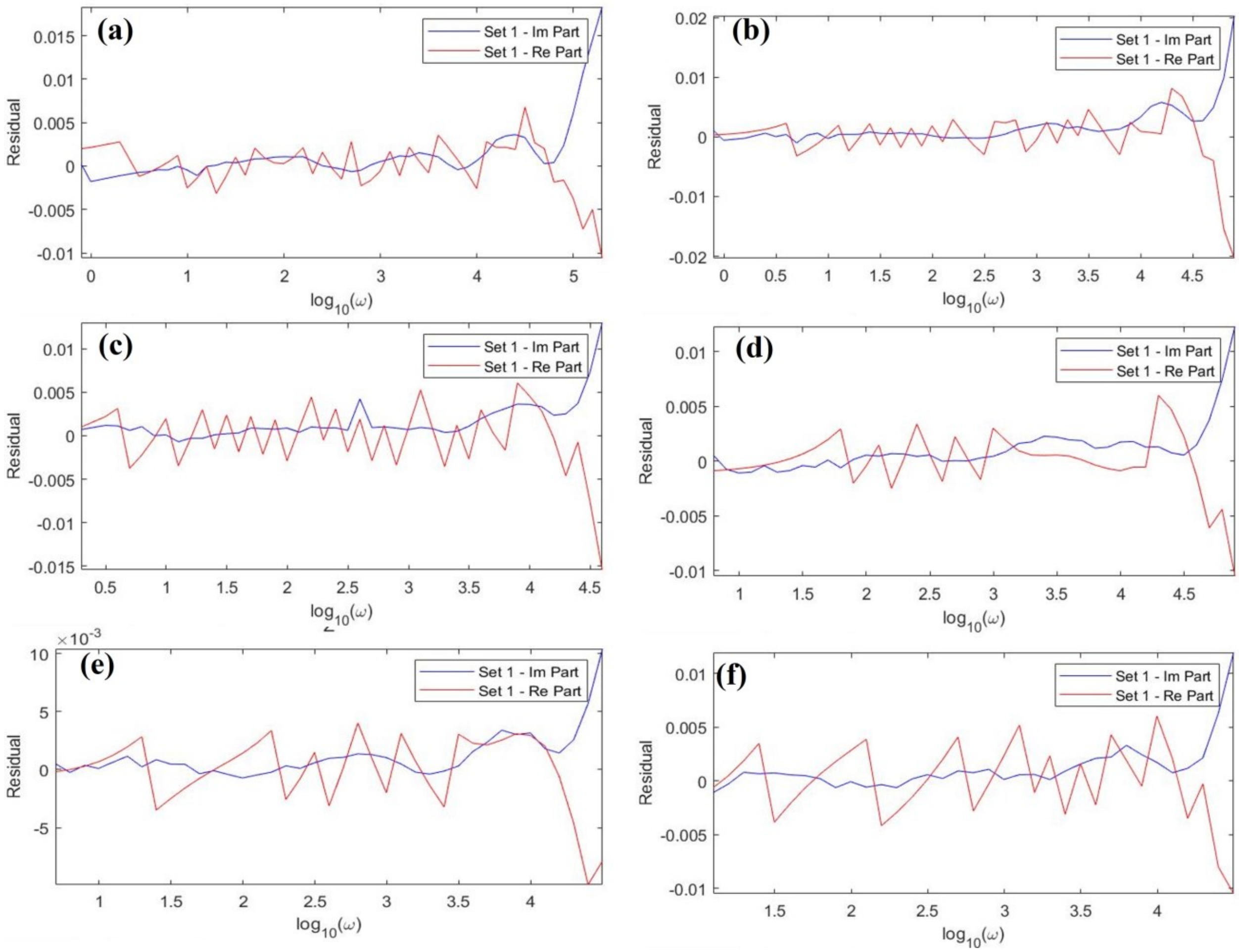

Figure S2. Residual Vs. log10(ω) plot for NSC (a-c) and PrO_x-NSC (d-f) impedance at 600 (a,d), 650 (b ,e) and 700 ºC (c,f).

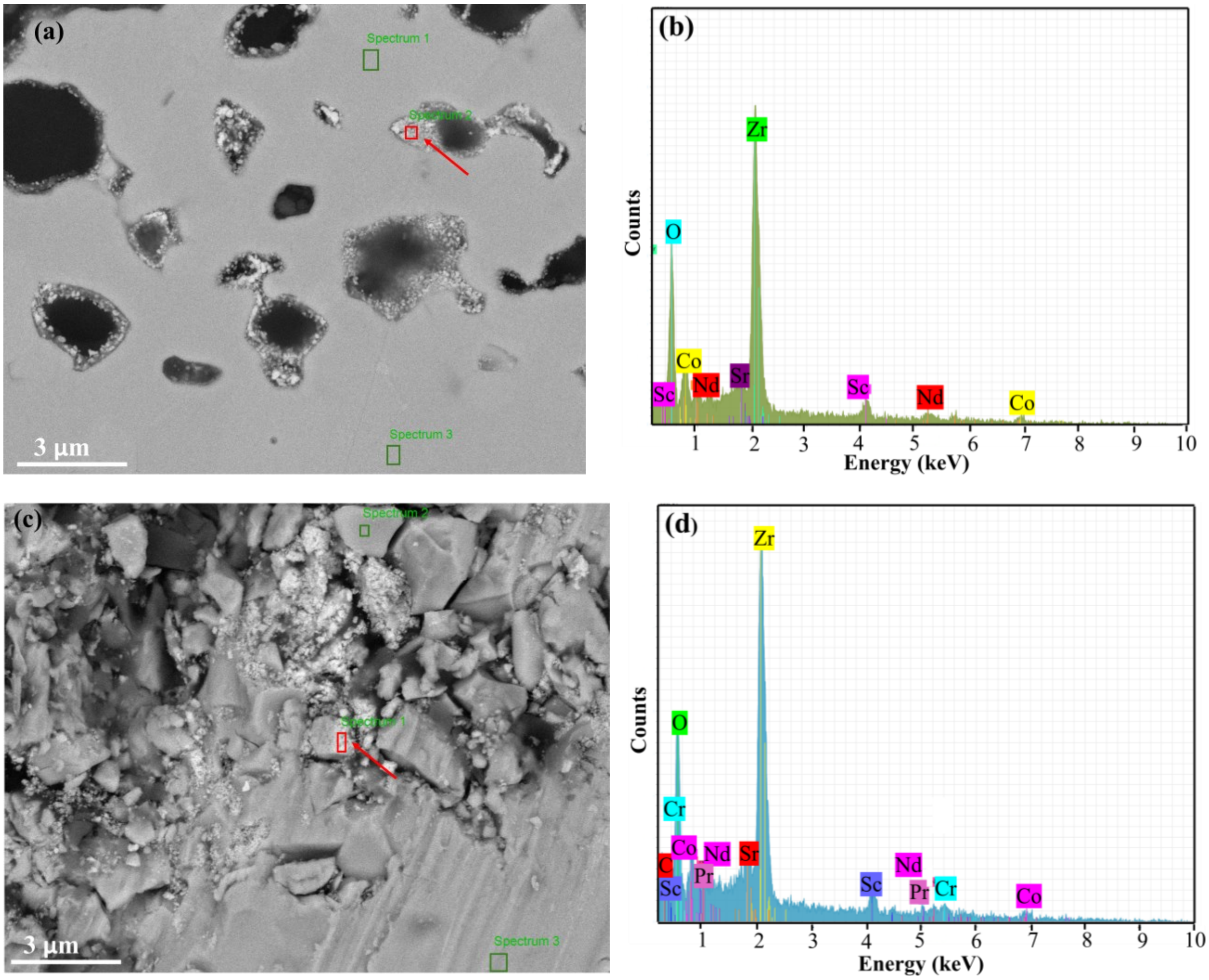

Figure S3. SEM images and EDX spectra of half cells infiltrated with (a,b) NSC (spectrum 2) and (c,d) PrOx-NSC (spectrum 1) after the test in air at 600-700 °C.

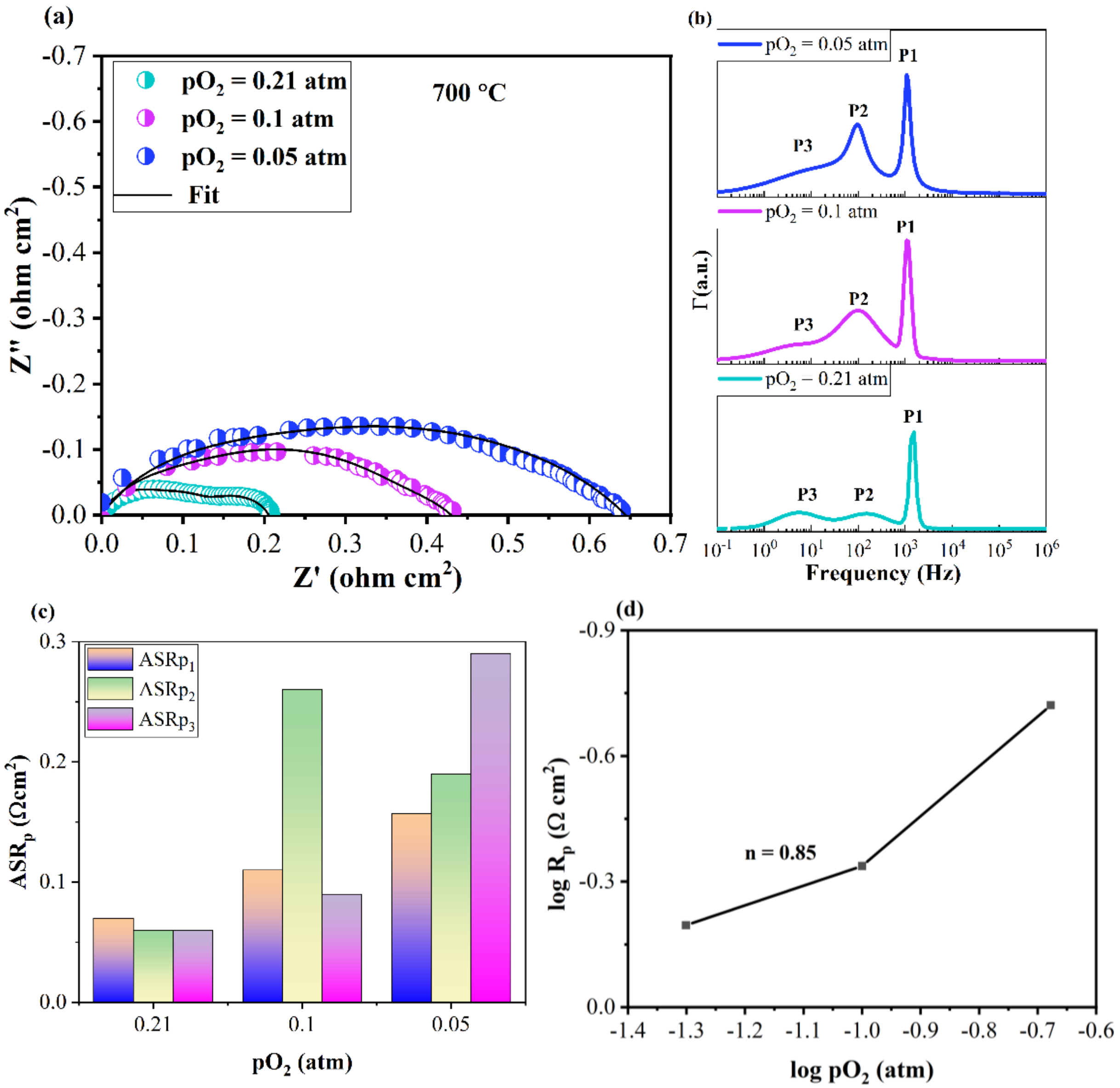

Figure S4. (a) The Nyquist plots and (b) the DRT peaks for NSC infiltrated cell at 700 °C in different oxygen pressure atmospheres; The corresponding (c) simulated resistances and (d) rate limiting steps of NSC.

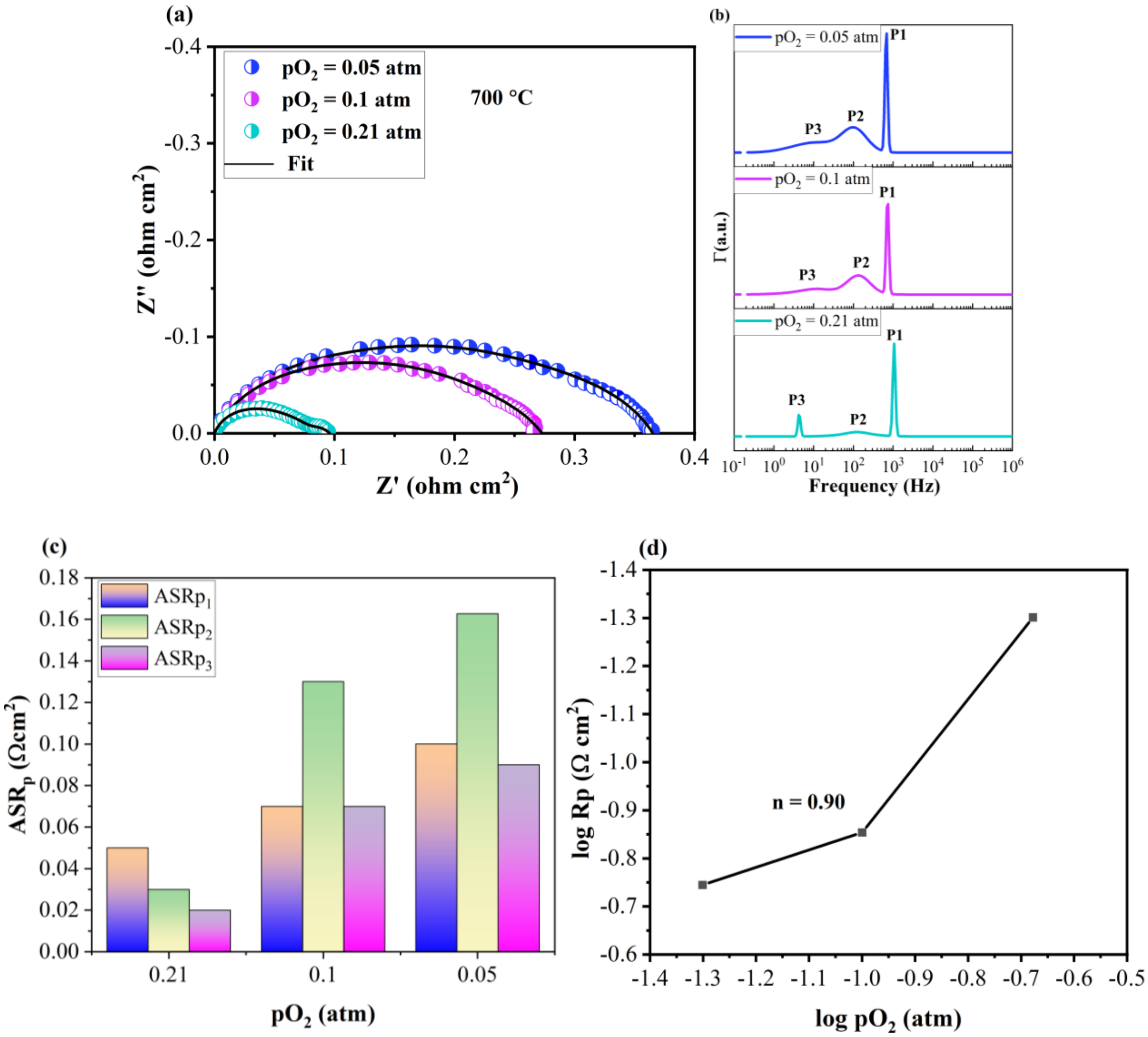

Figure S5. (a) The Nyquist plots and (b) the DRT peaks for PrO_x -NSC infiltrated cell at 700 °C in different oxygen pressure atmospheres; The corresponding (c) simulated resistances and (d) rate limiting steps of PrO_x -NSC.

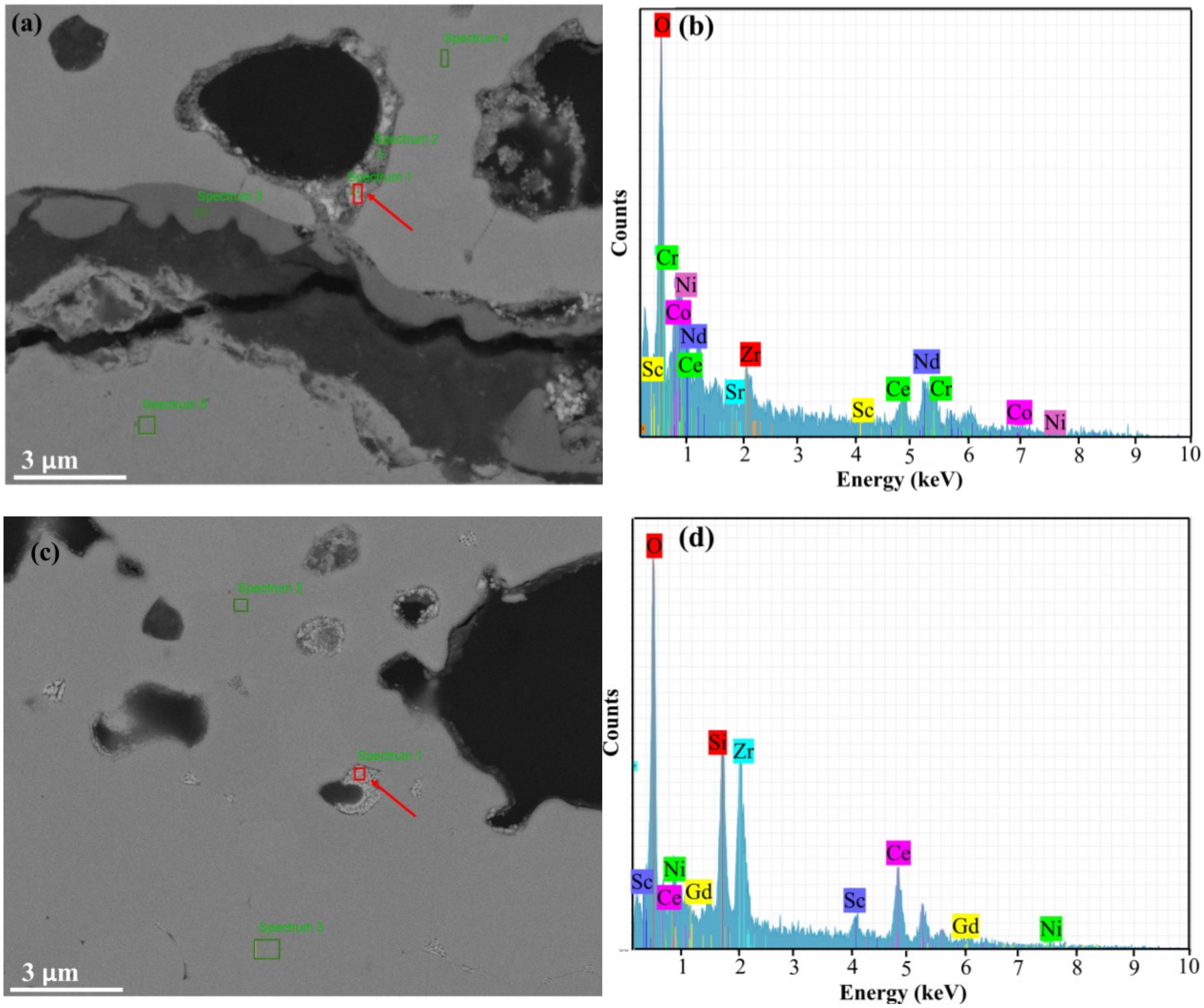

Figure S6. SEM images and EDX spectra of single cell infiltrated with (a,b) NSC (spectrum 1) on cathode side and (c,d) Ni-GDC (spectrum 1) on the anode side after the test in 3% H_2O/H_2 at 600-700 ℃.

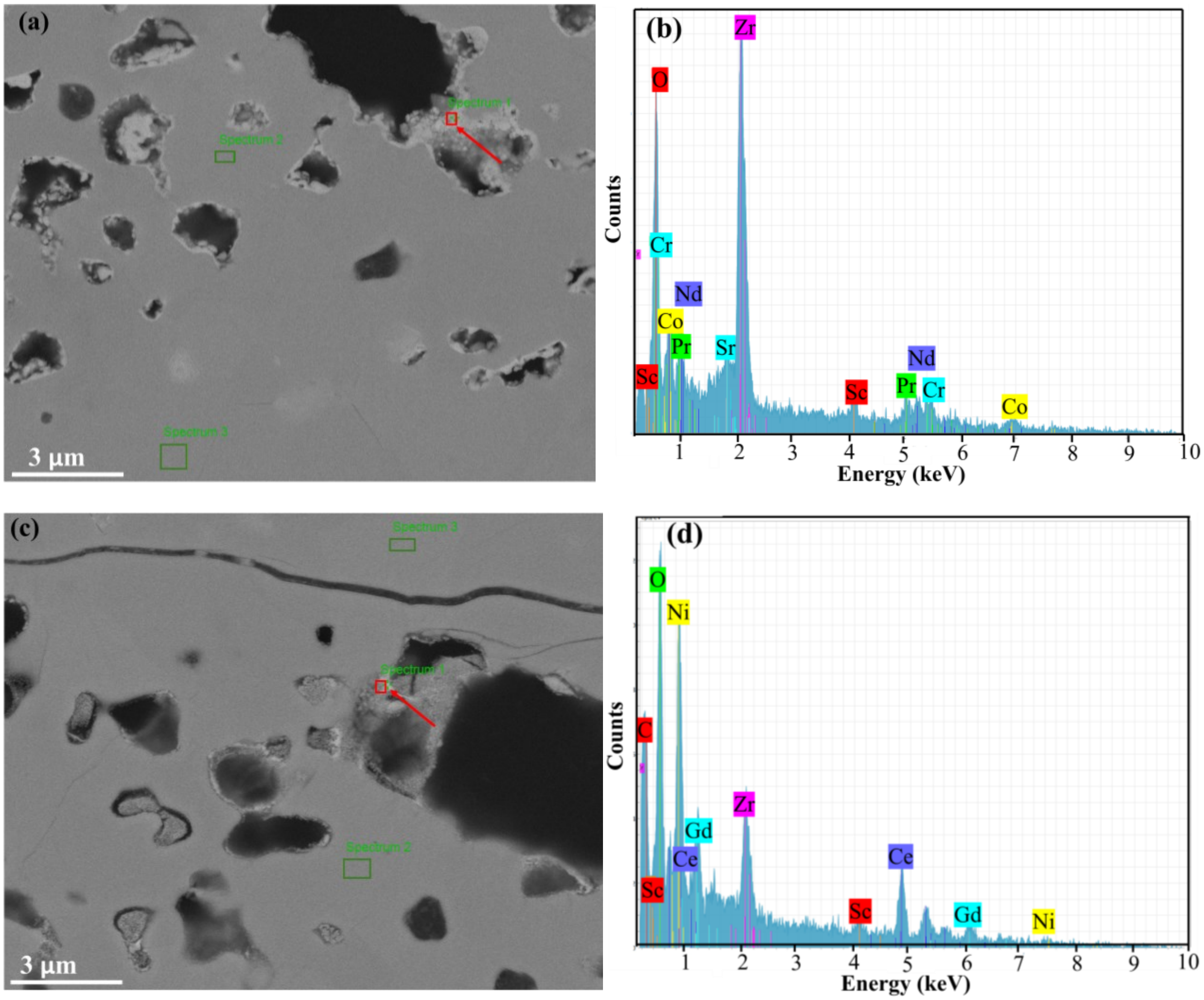

Figure S7. SEM images and EDX spectra of single cell infiltrated with (a,b) PrOx- NSC (spectrum 1) on cathode side and (c,d) Ni-GDC (spectrum 1) on the anode side after the test in 3% H_2O/H_2 at 600-700 °C.

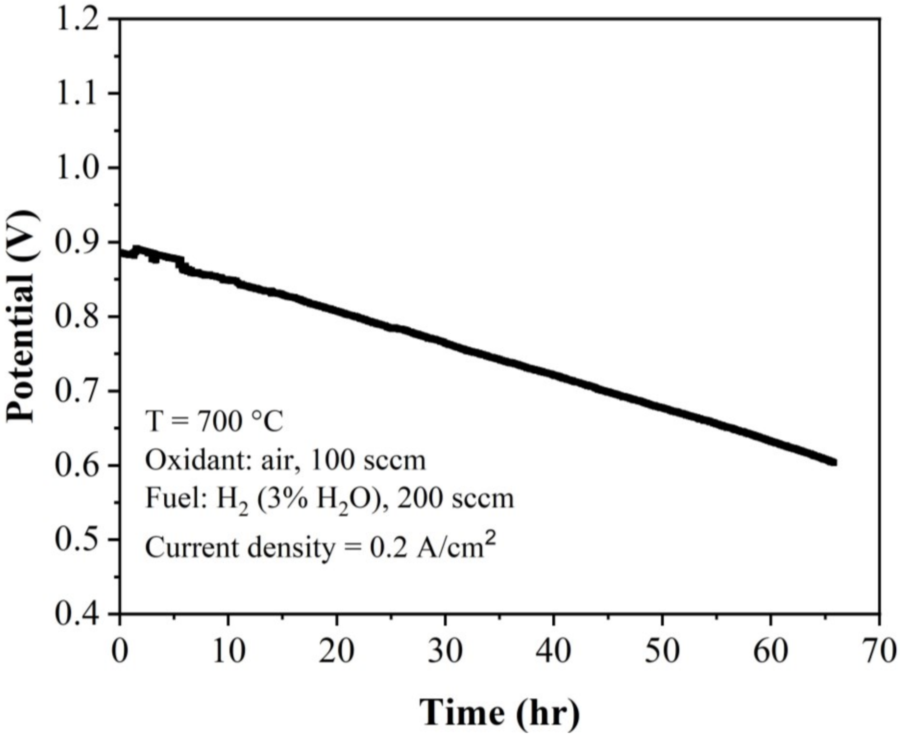

Figure S8. Stability of the single cell infiltrated with PrO_x-NSC under a constant voltage of 0.7 V at 650 °C for 65 h.
